# *Alternaria* keratitis after uneventful phacoemulsification in an otherwise healthy adult

**DOI:** 10.1186/s12348-016-0072-5

**Published:** 2016-02-16

**Authors:** Sana Khochtali, Amir Hriz, Fatma Abid, Imen Khairallah-Ksiaa, Bechir Jelliti, Moncef Khairallah

**Affiliations:** Department of Ophthalmology, Fattouma Bourguiba University Hospital, Faculty of Medicine, University of Monastir, Monastir, Tunisia

**Keywords:** Phacoemulsification, Keratitis, Fungi, *Alternaria*

## Abstract

**Background:**

Fungal infections of self-sealing corneal incisions in cataract surgery are scarce. We report a case of *Alternaria* keratitis, several weeks after uneventful clear-cornea phacoemulsification.

**Findings:**

A 42-year-old woman, with a history of retinitis pigmentosa, complained of painful red right eye, 45 days after uneventful self-sealing clear-cornea phacoemulsification. Slit-lamp examination revealed multiple snow-like contiguous stromal infiltrates, with irregular margins, and no epithelial defect. These infiltrates were unresponsive to topical quinolones and topical corticosteroids as well as oral valaciclovir. Culture from corneal biopsy specimen grew *Alternaria* species. Management consisted of topical amphotericin-B, and then a combination of topical and oral voriconazole. The corneal infiltrates progressively healed. One year later, the best-corrected visual acuity was 20/400.

**Conclusions:**

Fungal infection, particularly *Alternaria* keratitis, should be considered in the differential diagnosis of delayed post-cataract surgery keratitis. Prompt diagnosis and management are mandatory to improve visual prognosis.

## Findings

### Introduction

Infectious complications after cataract surgery are usually sight-threatening. They mainly include postoperative endophthalmitis, and more rarely wound infections which may be associated or not with endophthalmitis. There have been a few reports about wound infections after clear-cornea phacoemulsification. Bacteria are the most common causative agents [1]. Fungal infection of self-sealing corneal incisions in cataract surgery is scarce, with *Aspergillus* being the most commonly identifiable fungi in this setting [1, 2].

We herein report a case of *Alternaria* keratitis, 45 days after uneventful clear-cornea phacoemulsification in a 42-year-old otherwise healthy patient.

### Case report

A 42-year-old woman with a history of retinitis pigmentosa, pseudophakic in both eyes, was referred to our department with vision blurring and pain in the right eye (RE). Uneventful self-sealing clear-cornea phacoemulsification with in-the-bag foldable hydrophilic acrylic intraocular lens implantation had been performed in the RE, 45 days prior to the onset of symptoms. The main port had been temporal. Medical history ruled out ocular trauma and contact lens wear.

**Fig. 1:**
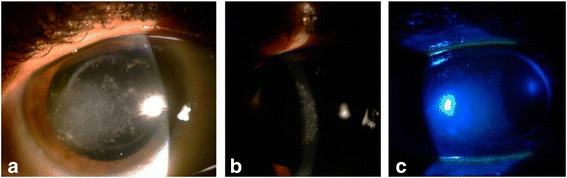
Slit-lamp photography at presentation. Slit-lamp photographs of the right eye showing multiple snow-like contiguous stromal infiltrates  (**a**, **b**) with no epithelial defect (**c**)

The initial best-corrected visual acuity (BCVA) was 20/1000 in the RE and 20/200 in the left eye. Slit-lamp examination of the RE revealed multiple snow-like contiguous stromal infiltrates, with irregular margins along with stromal edema (Fig. 1). These lesions extended from the temporal corneal periphery to the central cornea over 8 mm. The overlying epithelium was intact. Conjunctival hyperemia was mild. There was no anterior chamber reaction. Vitreous appeared clear. Results of the examination of the left eye were unremarkable, except for fundus features of retinitis pigmentosa. The nasolacrimal ducts were patent in both sides.

**Fig. 2:**
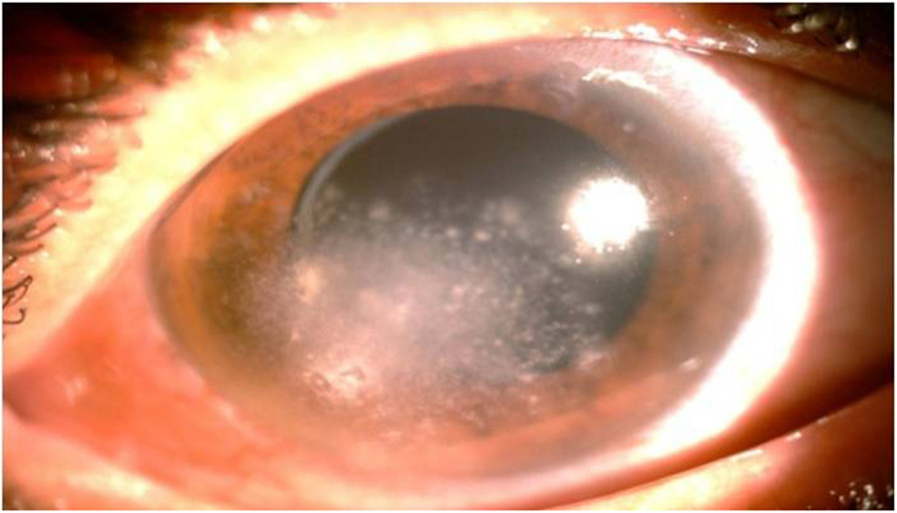
Slit-lamp photography before initiation of antifungal therapy. Slit-lamp photograph of the right eye, 4 weeks after onset of symptoms, showing extension of corneal lesions (after treatment with antibiotics, antivirals, and topical steroids)

**Fig. 3:**
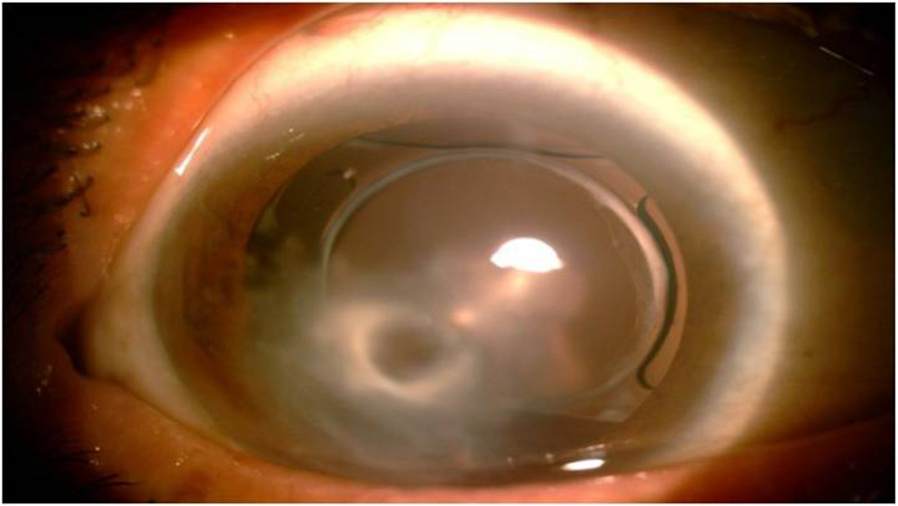
Slit-lamp photography at last follow-up. Slit-lamp photograph of the right eye, at 1-year follow-up, showing corneal opacity with central descemetocele

The patient had received ofloxacin drops for 15 days, before referral, without any response. Corneal scrapings over the stromal infiltrates did not reveal any organisms on microscopy or culture. Herpetic stromal keratitis was suspected, and the patient was treated with oral valaciclovir (2 g/day) and topical corticosteroids (one drop every 2 h, then progressive tapering), for 15 days. However, snow-like lesions continued to progress (Fig. 2). Therefore, a corneal biopsy was performed and revealed septate hyphae. Culture grew *Alternaria* species. The patient received topical amphotericin-B (5 mg/mL) every 2 h. No improvement was observed. On day 7, the patient was started on topical voriconazole 1 % every hour and oral voriconazole 200 mg twice daily. Topical antifungal drops were progressively tapered and maintained for a total duration of 60 days. The area of the stromal infiltrates was replaced by a diffuse corneal opacity, associated with central descemetocele (Fig. 3). The final BCVA in the RE was 20/400 at 1-year follow-up. The patient declined any further surgery.

This case report was approved by local ethics committee.

### Discussion

Wound infection of a self-sealing corneal incision in cataract surgery, defined by an infectious process starting from the incision area, is rare. Fungi are less commonly identified as causative agents than bacteria, with *Aspergillus* ranking first, followed by *Candida*, *Fusarium*, *Alternaria*, and *Scedosporium* which were identified in only few cases [1–5].

The sources of microorganisms in clear-cornea self-sealing incisions are probably similar to those of postoperative endophthalmitis. The possible sources include the patient’s eyelids and conjunctiva, contaminated instruments, intraocular lenses, or irrigating solutions, airborne germs, and major breaches in the sterile techniques [2, 3, 6]. Many factors have been involved in the development of self-sealing wound infections including immunosuppression, diabetes mellitus, dry eye syndrome, preoperative eyelid infections, obstructed nasolacrimal duct, prolonged use of systemic or topical steroids, and defect in wound architecture [2].

Fungal wound infections are rare. Garg et al. reported five cases of fungal infection of sutureless self-sealing incision for phacoemulsification and identified *Aspergillus* in four eyes (80 %) and *Candida* in one case (20 %). In our patient, culture of corneal biopsy grew *Alternaria*. It is a filamentous dematiaceous ubiquitous mold that is frequently isolated from soil, plants, and indoor air environment particularly in tropical and subtropical climates [3]. *Alternaria* keratitis has been rarely reported. It usually occurs after ocular trauma or in the setting of contact lens wear [7–10]. Postsurgical *Alternaria* corneal infection has been described after corneal transplantation [11], laser in situ keratomileusis [12], and after cataract surgery [3]. To the best of our knowledge, *Alternaria* keratitis has been previously described as a complication of a self-sealing clear-cornea phacoemulsification in only one patient [3]. Similarly to our patient, the fungal origin of the keratitis in this patient was initially overlooked. Delay in diagnosis was 5 months in the previously reported case, and 1 month in our case. The use of topical steroids has worsened the infectious process in both cases. Early recognition of the snow-like appearance of the infiltrates associated with satellite lesions as seen in our case, or of fernlike feathery margins could have allowed an earlier diagnosis of the fungal cause of infectious keratitis [3, 8].

However, interval between the phacoemulsification procedure and the insidious onset of symptoms was longer in our patient (45 versus 15 days). The source of the wound infection remained unknown in both cases. Our patient was otherwise healthy, unlike the previously reported patient who had a history of diabetes [3]. In fact, diabetes mellitus has been described as an important predisposing or aggravating factor in fungal wound infections [2].

*Alternaria* wound infections, similarly to other fungal wound infections, are often deep-seated, and superficial corneal scraping often yields negative results. Repeated deep scrapings, corneal biopsy as in our patient, or anterior chamber tap when it is compromised usually allow the identification of the causative germ [2, 3]. Polymerase chain reaction can be a useful adjunct to smear and culture in the diagnosis of fungal keratitis, in cases of failed detection with routine techniques [13].

In vivo confocal microscopy may show hyperreflective filaments, corresponding to filamentous fungi including *Alternaria*, and thus may help to establish an early diagnosis [9]; however, this technique is not widely available. In addition to that, anterior segment optical coherence tomography may visualize fungal hyphae growing into anterior chamber from the cornea, indicating the filamentous fungal origin of keratitis [8, 9].

In *Alternaria* keratitis, drops of fluconazole 0.02 %, voriconazole 1 %, ketoconazole, and amphotericin B maintained for several weeks are helpful to control infection [3]. Topical caspofungin 0.5 % associated with intrastromal voriconazole was used in a refractory case [7]. In our patient, improvement was not seen with amphotericin B, but significant improvement was observed soon after topical and systemic voriconazole was initiated.

In summary, *Alternaria* keratitis is a rare complication of self-sealing corneal incision phacoemulsification. It should be suspected when onset of keratitis is delayed and insidious. Satellite lesions and fernlike margins are very typical clinical features of filamentous fungal abscesses, including *Alternaria* keratitis. Prompt diagnosis and management are mandatory to improve visual prognosis.

## Abbreviations

BCVA: Best-corrected visual acuity
RE: Right eye
